# Supplementary material for: Chronically implantable μLED arrays for optogenetic cortical surface stimulation in mice
Source: Nat Commun. 2026 Jan 14;17:878. doi: 10.1038/s41467-025-68191-5 (PMC12827276; doi:10.1038/s41467-025-68191-5)
Supplement: Supplementary file 1 — Supplementary Information [file 41467_2025_68191_MOESM1_ESM.pdf]

Supplementary materials for the paper titled:

Chronically implantable µLED arrays for optogenetic cortical surface stimulation in mice

Ryan Greer[1†*], Antonin Verdier[2†], Emma Butt[1], Yunzhou Cheng[1], Ella Callas[2], Niall McAlinden[1], Alicia Aniorte[2], Eya Mabrouk Kakaouia[2], Magdalena Pereyra[2], Martin D. Dawson[1], Brice Bathellier[2‡] & Keith Mathieson[1‡*]

Affiliations:

[1]University of Strathclyde, Institute of Photonics, Glasgow, G1 1RD, UK
[2]Université Paris Cité, Institut Pasteur, AP-HP, INSERM, CNRS, Fondation Pour l'Audition, Institut de l'Audition, IHU reConnect, F-75012 Paris, France
[†]These authors contributed equally
[‡]Joint senior authors
[*]Corresponding authors; contact at ryan.greer@strath.ac.uk or keith.mathieson@strath.ac.uk

Reference	Pixel size	No. pixels	Pixel pitch	Stimulation area	Power density dynamic range on brain surface (mW mm^{-2})	Animal model
Pollmann et al. 2024	0.2 x 0.1 mm^2	48	0.6 – 1.25 mm	6.4 x 7.8 mm^2	70	NHP
Rajalingham et al. 2021	0.5 x 0.5 mm^2	24	1 mm	5 x 5 mm^2	56	NHP
Shin et al. 2024	1.6 x 1.6 mm^2	4	>1 mm	>4 mm^2	-	Mouse (transcranial)
Komatsu et al. 2017	0.33 x 0.33 mm^2	8	4 mm	8 x 16 mm^2	-	NHP
Ohta et al. 2021	0.35 x 0.28 mm^2	48	1 mm	9 x 6.5 mm^2	>10	NHP
Lee et al. 2018	0.05 x 0.05 mm^2	12	0.5 mm	2 x 1.5 mm^2	>10	Rat
This work	0.04 x 0.04 mm^2	100	0.2 mm	2 x 2 mm^2	>50	Mouse

Supplementary Table 1: Comparison of chronically implantable μLED/LED array cortical surface stimulators.

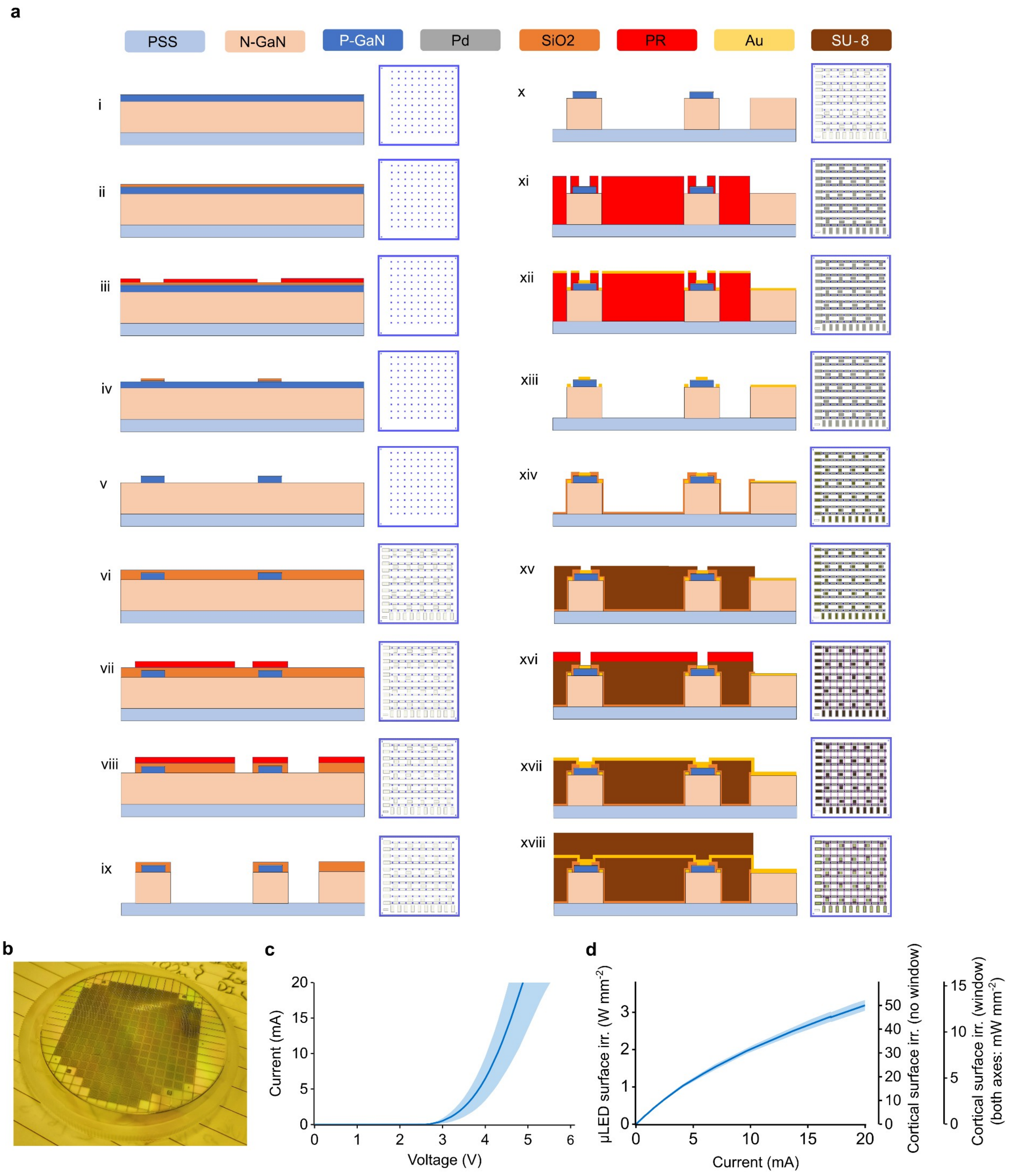

Supplementary Figure 1: Summary of μLED array fabrication process and characterisation. (a) Cross-sectional view of fabrication steps (left), corresponding photolithography mask for each fabrication step (right). **(b)** 2-inch wafer-scale fabrication with 220 μLED arrays. **(c)** Typical current-voltage (I-V) characteristic. **(d)** Typical irradiance-current (L-I) characteristic, at μLED and cortical surface with and without a cortical window present. Both (c) and (d) show mean and standard deviation of n = 10 μLEDs.

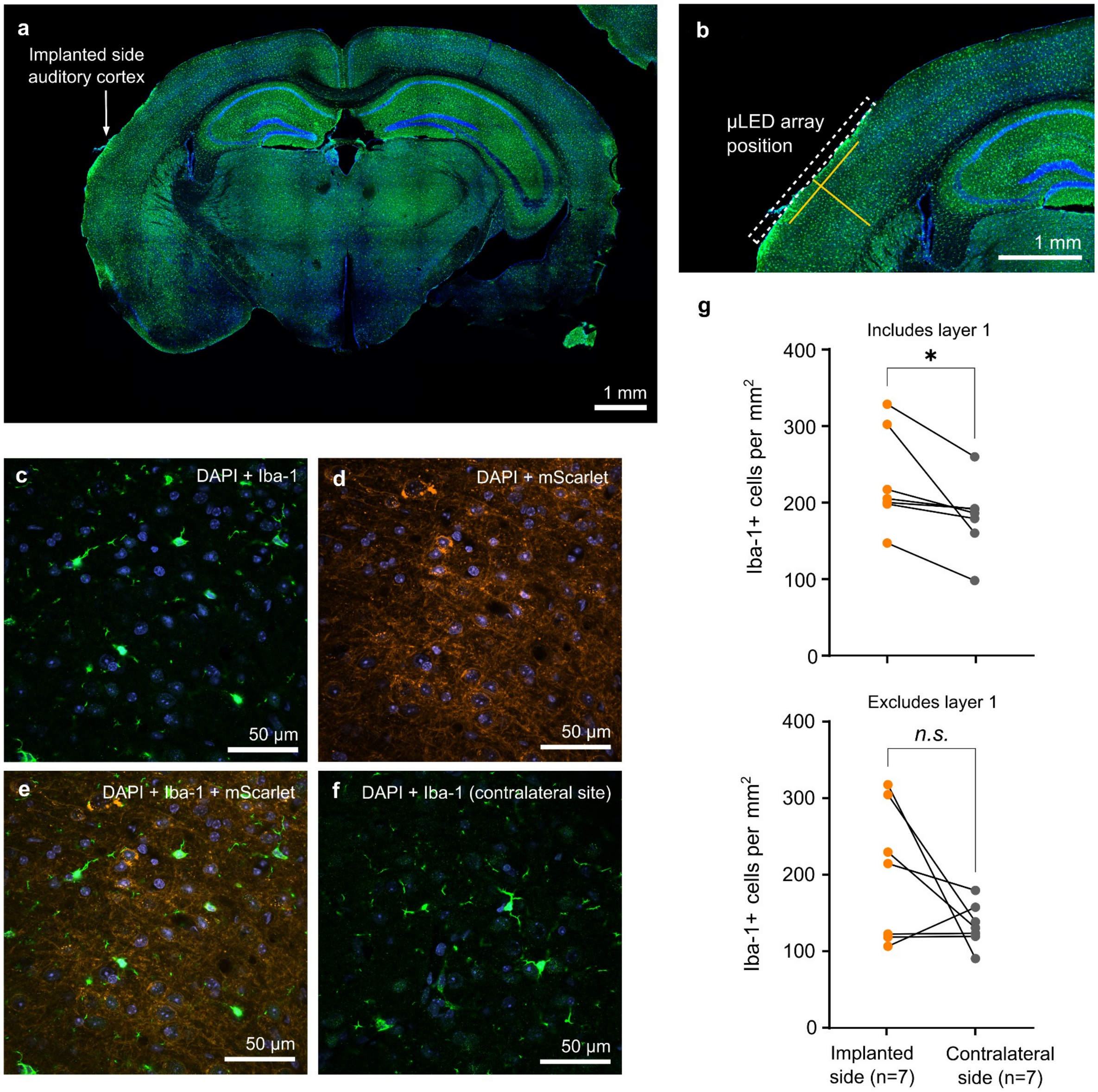

Supplementary Figure 2: Histological analysis. (a) Coronal view of brain slice stained with DAPI and Iba-1, with auditory cortex on implanted side indicated. **(b)** Zoomed in view of implanted auditory cortex; μLED array geometry shown as dashed outline; layer 1-2/3 boundary and depth of analysed region indicated by solid yellow lines. **(c)** Zoomed example of implanted region analysed to examine immune response (staining: DAPI + Iba-1). **(d)** Same region as (c) (DAPI + mScarlet). **(e)** Same region as (c) and (d) (DAPI + Iba-1 + mScarlet). **(f)** Similar example region for contralateral side (DAPI + Iba-1). **(g)** Immune response as number of Iba-1+ cells (microglia) per square millimetre of imaged tissue, comparing both implanted and contralateral sides across n = 7 coronal slices for 3 mice; non-parametric Wilcoxon matched-pairs signed rank test. *indicates statistical significance; n.s. indicates not significant. Layer 1 was removed from both sides to exclude surface immune response from the analysis (bottom plot).

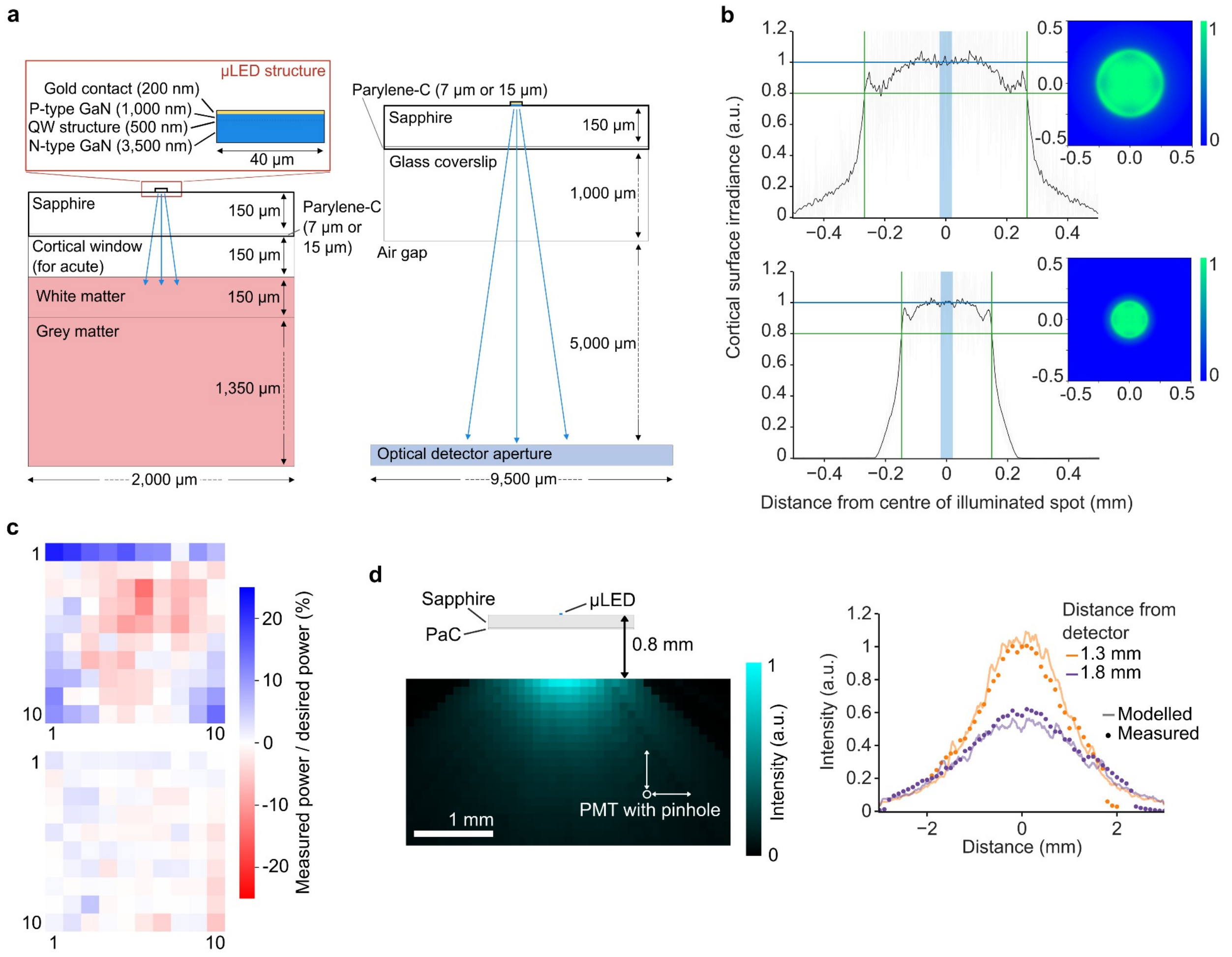

Supplementary Figure 3: Supplementary optical modelling. (a) Schematic of optical model used to perform Monte Carlo simulations of blue light from µLED array in brain tissue (left); schematic of optical model representing laboratory setup for device calibration (right). 7 µm parylene-C (PaC) modelled for acute device (with cortical window) and 15 µm PaC modelled for chronic device (implanted directly on brain tissue). **(b)** Central cross-section of irradiance of illuminated spot on the cortical surface with estimated spot diameter, for acute *in-vivo* experiment with window (top) and chronic implantation without window (bottom). Insets show full 2D irradiance map over 1x1 mm^2 detector. **(c)** Normalised measured cortical irradiance across device for the optimal fixed drive current (top) and for drive currents individually inferred for each µLED from optical models (bottom). **(d)** Emission profile of single µLED through air, measured using a PMT detector with pinhole maneuvered through emission profile (100 µm measurement spacing) (left). Emission profile at different depths compared with cuts at equivalent distance from an optical model of device in air (right).

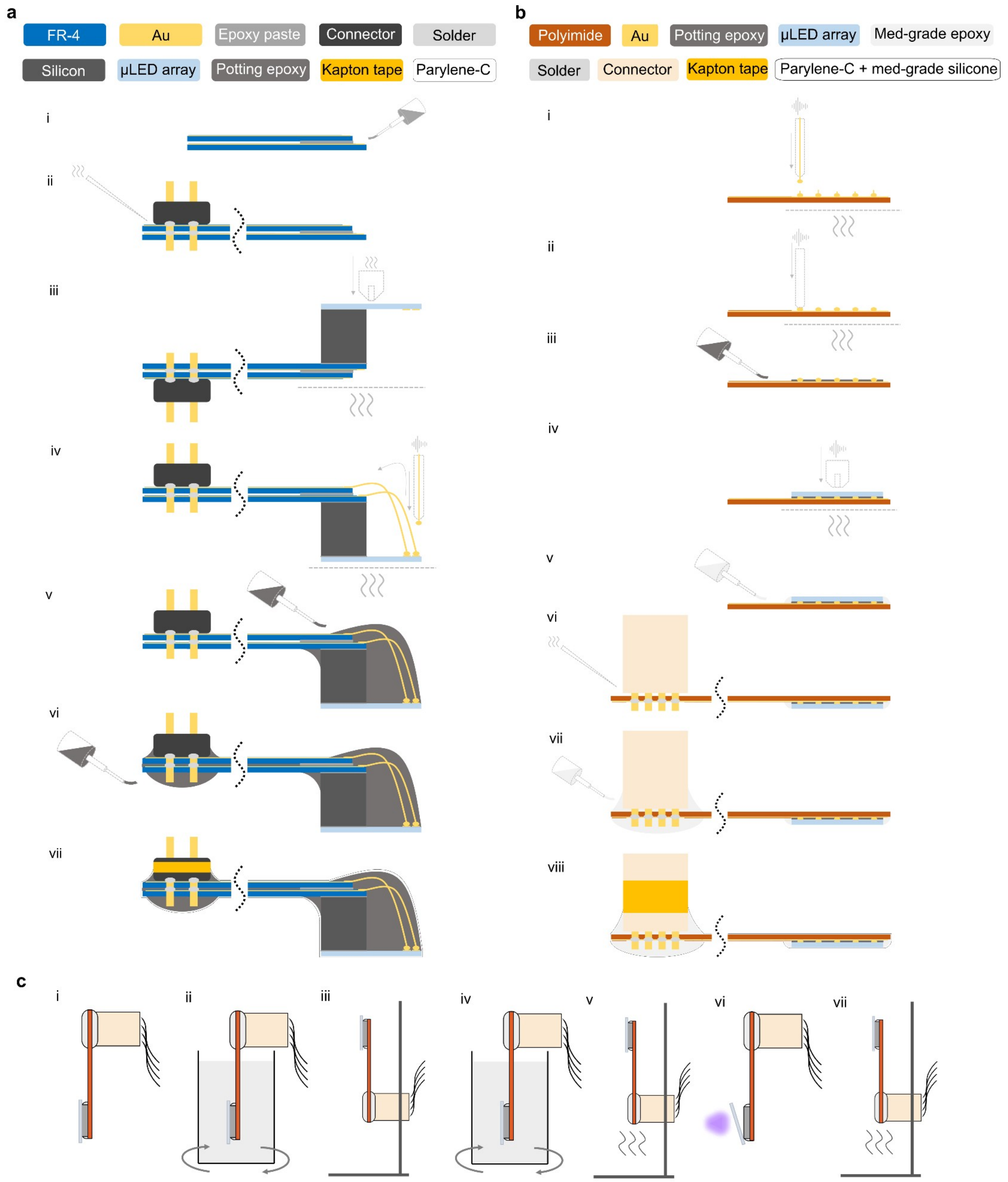

Supplementary Figure 4: Cross-sectional view of integration process for rigid (acute) and flexible (chronically implantable) systems. (a) Bonding two 2-layer 200 µm thin FR4 PCBs (i); soldering 20-pin linear connector (ii); Bonding spacer block and µLED array using flip-chip bonding (iii); gold ball-wedge wire bonding of µLED array to PCBs (iv); potting wire bonds (v); potting connector and space between PCBs (vi); parylene-C coating, connector sealed with kapton tape before (vii). **(b)** Bump-bonding gold balls to flexible 100 µm thin polyimide PCB (i); coining bump bonds to remove tail (ii); underfill of bonding area prior to flip-chip bonding (iii); thermosonic flip-chip bonding of µLED array to flexible PCB (iv); medical-grade epoxy bevelled underfill around µLED array (v); soldering Omnetics connector (vi); potting Omnetics connector with medical-grade epoxy (vii); parylene-C and medical-grade silicone dispersion coating; connector sealed with kapton tape before parlyene-C coating and sapphire surface protected with UV-sensitive dicing tape prior to silicone dip coating and removed after silicone is partially cured (viii). **(c)** Steps for medical-grade silicone dip coating encapsulation. Applying UV-sensitive dicing tape (i). Dip 1 + beaker rotation (ii). Dry 1 (10 mins, room temp) (iii). Dip 2 + beaker rotation (iv). Dry 2 (1 hour, 50 °C) (v). UV exposure and peel back dicing tape (vi). Final dry (150 °C, until rubber) (vii).

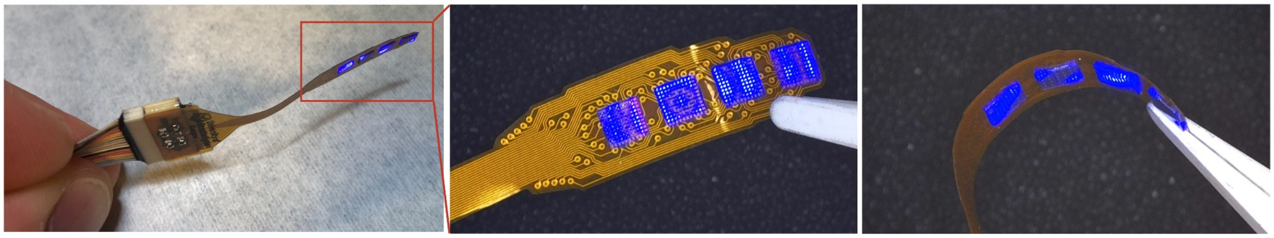

Supplementary Figure 5: Flexible, chronically implantable device integrating 4 µLED arrays arranged in a line, separated by approx. 1 mm gaps.

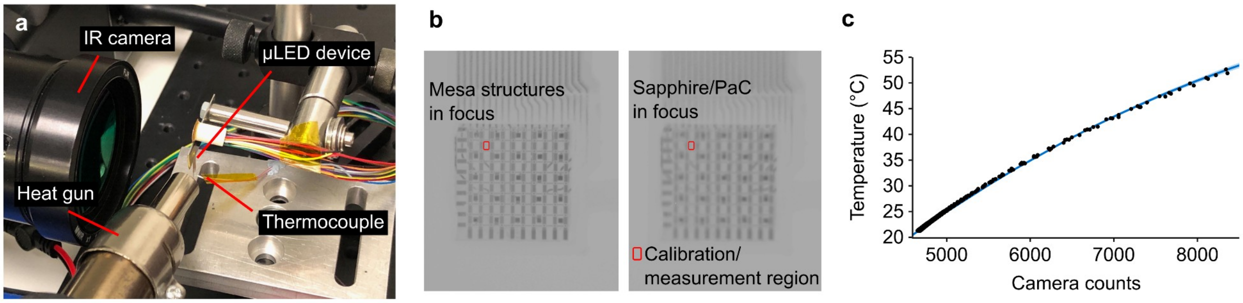

Supplementary Figure 6: Supplementary figures on thermal imaging. (a) Photograph of thermal imaging experiment in laboratory showing infrared (IR) camera imaging sapphire surface. Thermocouple is used to measure actual temperature during calibration. **(b)** IR image showing locations used for thermal measurement and calibration, taken as an average within rectangular area. **(c)** Quadratic regression fitted to thermocouple data against camera counts; every tenth data point is shown.

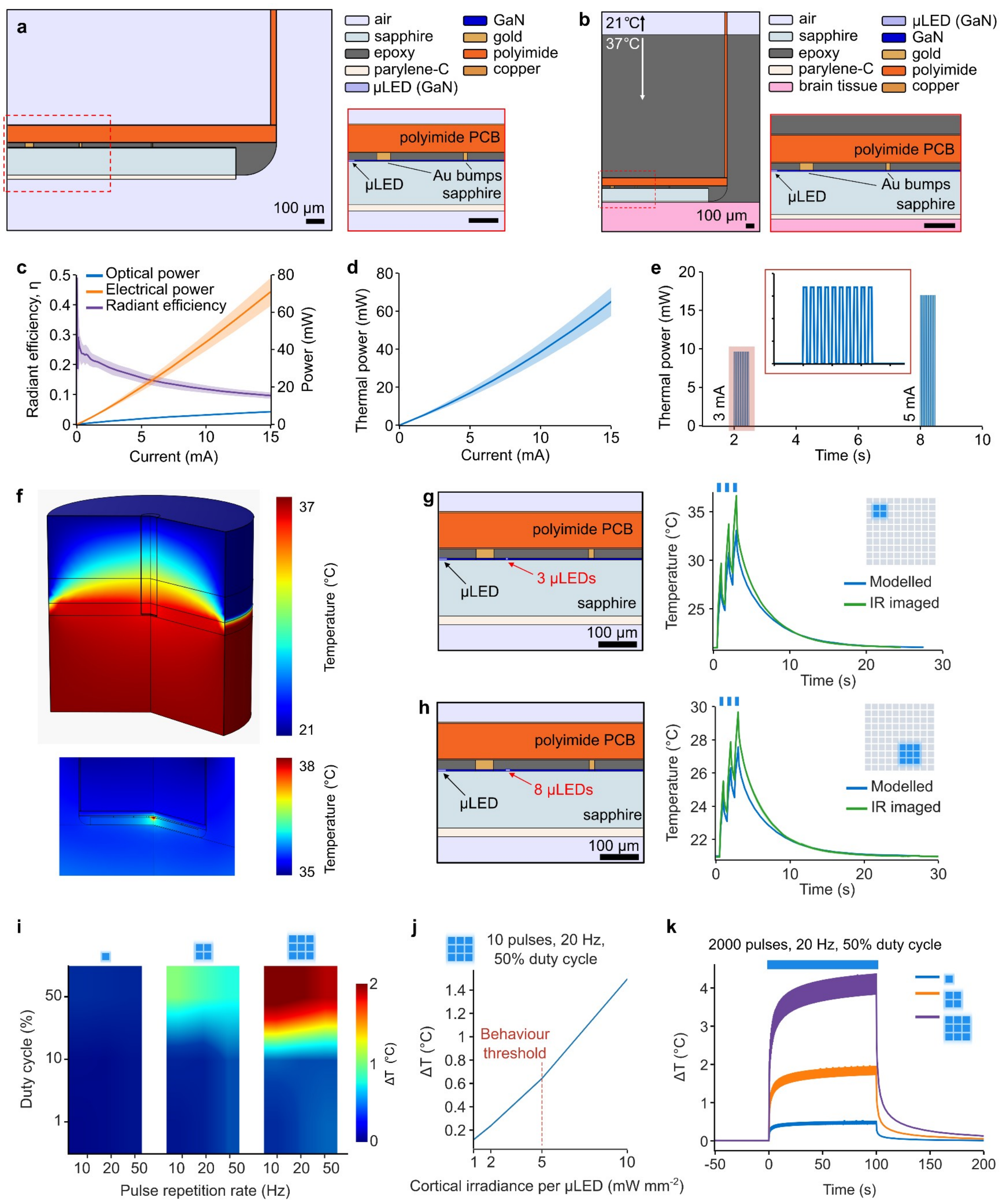

Supplementary Figure 7: Supplementary figures on thermal modelling. (a) Schematic of thermal model used for direct comparison with infrared (IR) imaging experiment. **(b)** Schematic of thermal model used for direct inference of thermal effects *in-vivo*. **(c)** Measured electrical and optical power (actual optical power generated within µLED was determined through optical models) and radiant efficiency as a function of drive current. **(d)** Thermal power as function of drive current. Both (c) and (d) show mean ± standard deviation for n = 10 µLEDs. **(e)** Thermal pulse protocol input to thermal model for a single µLED. **(f)** Example of 3D model from COMSOL after rotation about axis of symmetry. **(g)** Comparison of IR imaging with equivalent thermal model for 2x2 square of µLEDs simultaneously illuminated; protocol is 1 Hz, 50% duty cycle, 3 mA per µLED. **(h)** Panel equivalent to (g) but for a 3x3 square of µLEDs, 1 mA per µLED. **(i)** Evaluation of modelled peak temperature increase on brain surface, over 500 ms stimulation time, across different pulse repetition rates and duty cycles; interpolation is used on colour map between 9 data points per pattern. Modelled thermal power corresponds 3 mA equivalent per µLED. **(j)** Modelled peak temperature increase as a function of cortical irradiance; equivalent currents per µLED are (0.19, 0.38, 1.02, 2.26) mA. **(k)** Modelled temperature increase on brain surface over prolonged operation (100 seconds); modelled heat protocol (20 Hz, 50% duty cycle), with simulated thermal power equivalent to a drive current of 3 mA per µLED.

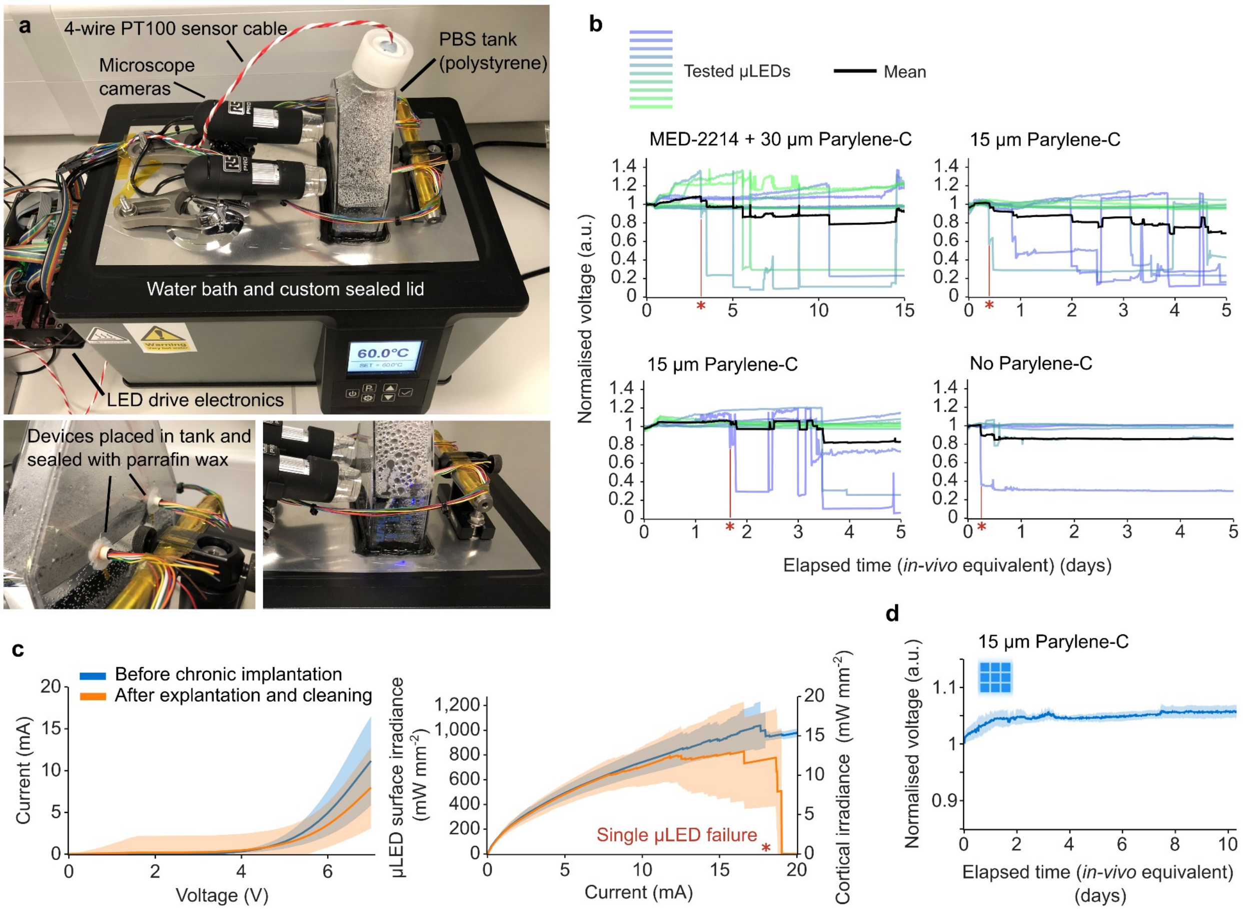

Supplementary Figure 8: Supplementary figures on accelerated ageing experiments and evaluation of chronically implanted devices. (a) Photograph of laboratory setup showing device submerged in phosphate-buffered saline (PBS) solution heated to 60 °C using a water bath with custom lid and tank. **(b)** Normalised measured voltage during accelerated ageing for different device encapsulations; experimental protocol same as in Fig. 2d. **(c)** I-V (left) and L-I (right) before chronic implantation for 29 days, and after behavioural experiments and explantation of a single device. Mean ± standard deviation for n = 100 µLEDs. **(d)** Normalised measured voltage during accelerated ageing for single 3x3 µLED pattern; experimental protocol same as in Fig. 2d with 2 mA per µLED. Mean and standard deviation of voltage measurements across 3 columns of 3 µLEDs in parallel.

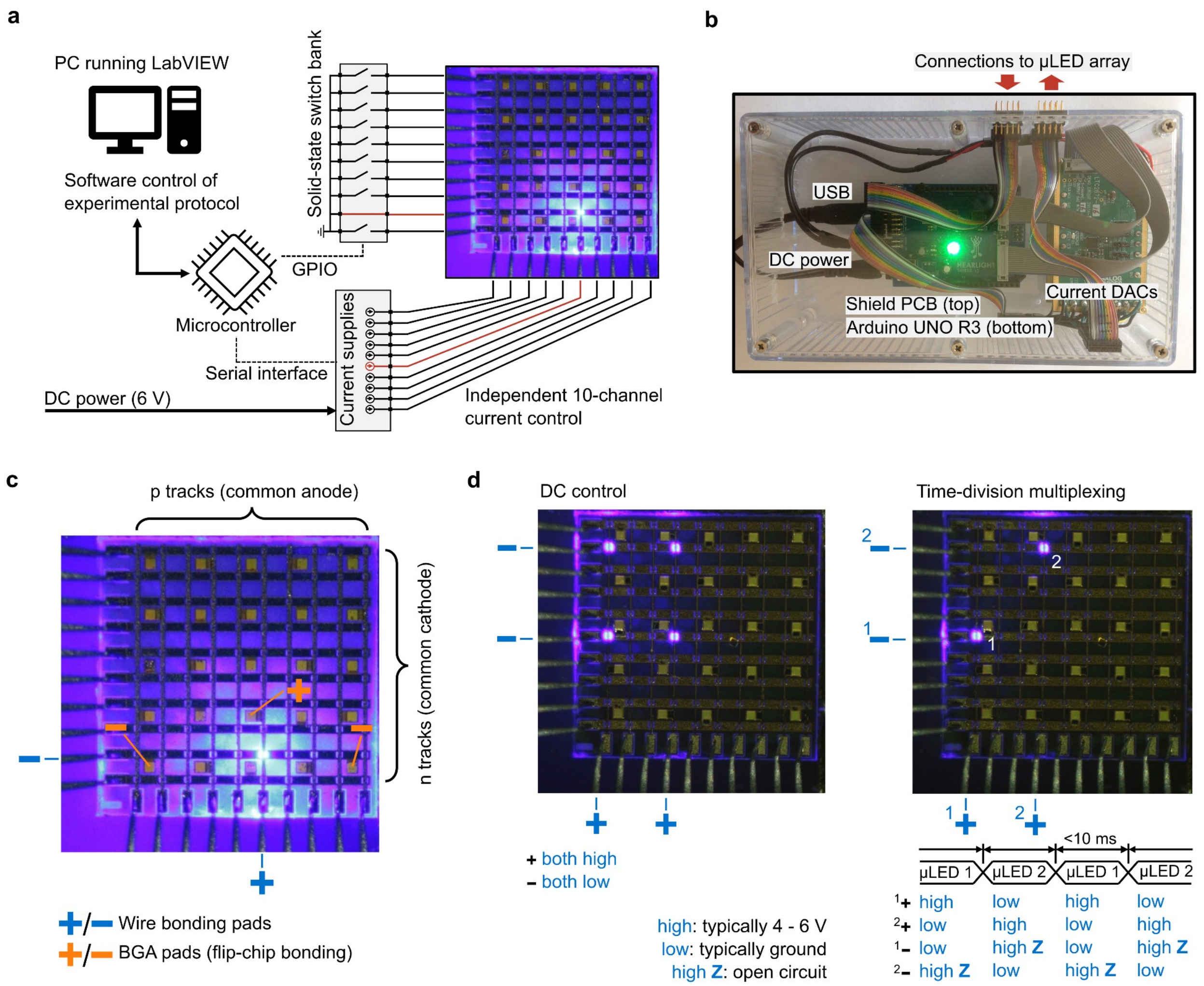

1+	high	low	high	low
2+	low	high	low	high
1–	low	high Z	low	high Z
2–	high Z	low	high Z	low

Supplementary Figure 9: Electronic controller for µLED arrays. (a) Schematic of custom electronic control system based on current drivers operated with a microcontroller and LabVIEW interface. **(b)** Annotated photograph of the portable boxed system. **(c)** Illustration of matrix addressing of a single µLED through wire bonding or ball-grade array (BGA) pads. **(d)** Illustration of operating modes; DC which limits pattern selection (left), and time-division multiplexing allowing arbitrary patterns to be displayed, albeit at a duty cycle (right).

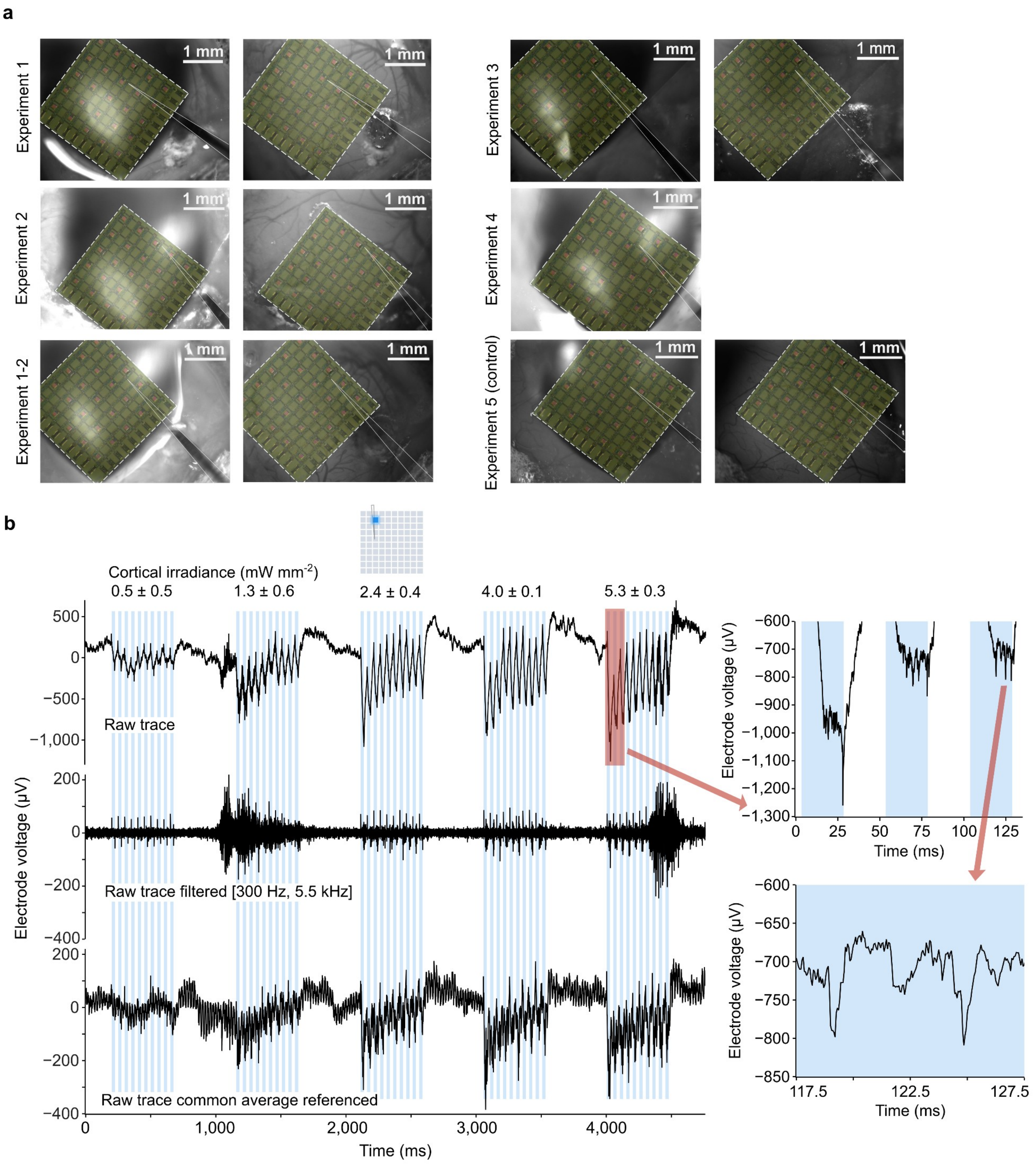

Supplementary Figure 10: Supplementary micrographs and recorded waveform from electrophysiology experiments. (a) Micrographs of craniotomy for each experiment with position of linear electrode array indicated relative to the placement of the μLED array. **(b)** Electrophysiology waveforms from a single electrode, corresponding to same μLED and trial as Fig. 3c. Top: raw trace; middle: raw trace after filtering with bandpass FIR filter (cutoffs 300 Hz and 5.5 kHz); bottom: raw trace after common median reference subtracted (no filtering). Insets show zoom of raw, unfiltered trace showing spiking waveforms induced by optogenetic stimulation.

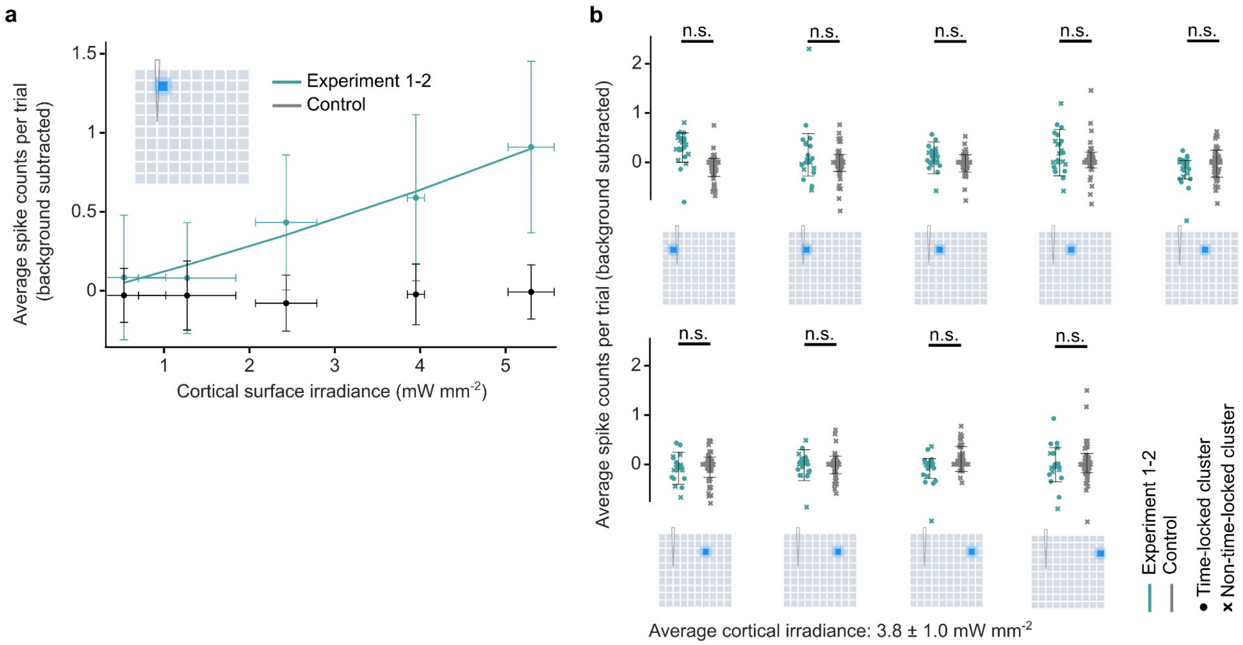

Supplementary Figure 11: Excluded electrophysiology experiment. (a) Dose response for Experiment 1-2 which was omitted from main analysis due to poor recording quality, showing average spike counts per trial with background subtracted under stimulation from same µLED as Fig. 3c and Fig. 3d. Vertical error bars show pooled mean and pooled SEM (n = 12 time-locked single units for optogenetic (ChR2+) experiment; n = 51 single units for control (ChR2-) experiment); horizontal error bars show estimated cortical irradiance delivered by µLED (mean ± standard deviation). **(b)** Average spike counts per trial with background subtracted, under stimulation from different µLEDs. Error bars show pooled mean ± pooled SEM (ChR2+: n = 23, ChR2-: n = 51 single units; time-locked single units are shown as circles, non-time-locked as x's). Significance levels determined using the independent two-sample, single-tailed t-test with alt. hypothesis: spike counts (ChR2+) > spike counts (ChR2-); n.s. means alt. hypothesis rejected at $p < 0.05$); exact p values given in Supplementary Table 2. Average cortical irradiance given as pooled mean ± pooled standard deviation.

μLED	Experiment 1	Experiment 2	Experiment 3	Experiment 4	Experiment 1-2
P2N3	0.0010	0.0075	0.00001	0.1523	0.1262
P3N3	0.0002	0.0154	0.0001	0.0295	0.3638
P4N3	0.0001	0.0062	0.00001	0.0744	0.3786
P5N3	0.0003	0.1491	0.0001	0.1730	0.3812
P6N3	0.0993	0.4576	0.2169	0.4049	0.6426
P7N3	0.0927	0.3274	0.1998	0.2821	0.5188
P8N3	0.2462	0.3968	0.0575	0.4149	0.5037
P9N3	0.3854	0.4491	0.7084	0.6005	0.7247
P10N3	0.1009	0.5214	0.1564	0.5102	0.5311

Supplementary Table 2: p values for statistical tests in Fig. 3f and Supplementary Fig. 11b. Independent two-sample, single-tailed Student's t-test with alt. hypothesis: spike counts (ChR2+) > spike counts (ChR2-); ChR2+ (optogenetic): Exp. 1 n = 18, Exp. 2 n = 45, Exp. 3 n = 16, Exp. 4 n = 53, Exp. 1-2 n = 23; ChR2- (control): n = 51 neurons.

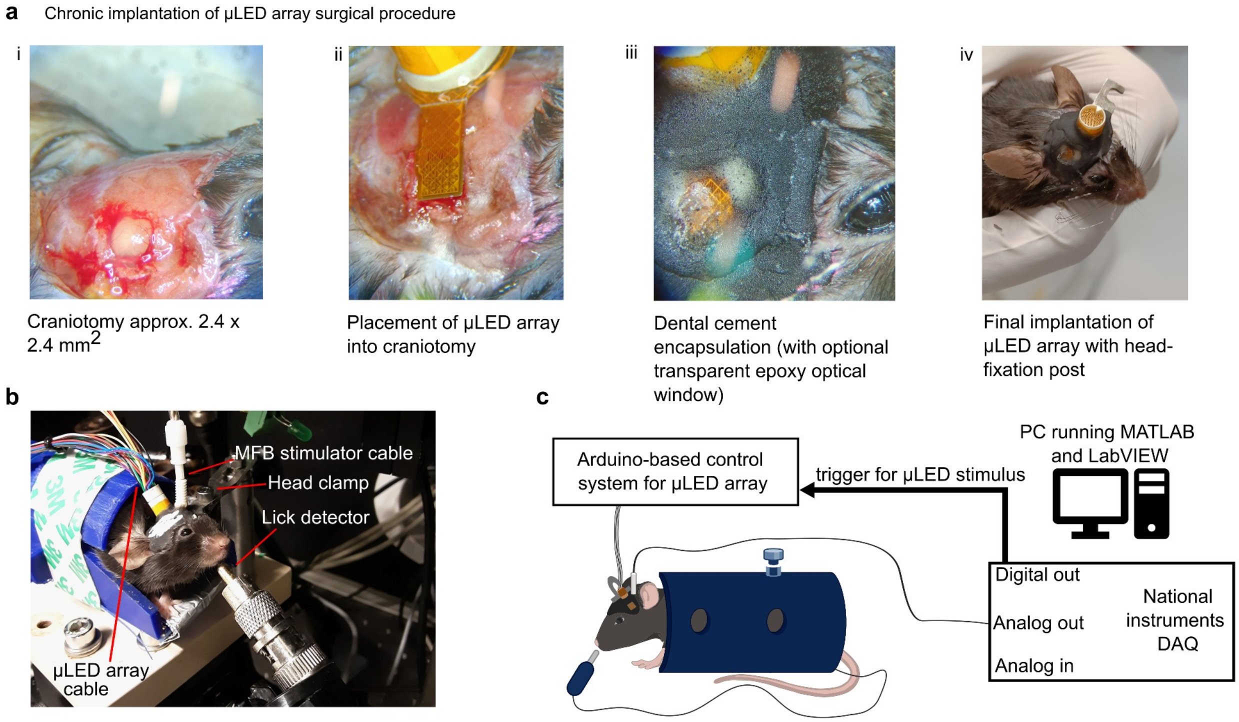

Supplementary Figure 12: Surgical steps for chronic implantation of µLED array and behavioural experimental setup. (a) Photographs of surgical steps for chronically implanting the µLED array with flexible packaging. A square-shaped craniotomy is performed and the dura mater removed. The µLED array is placed into the craniotomy and secured to the skull using dental cement. **(b)** Annotated photograph of head-fixed mouse within behavioural experimental setup. **(c)** Schematic drawing of behavioural experimental setup. The experiment is managed using a MATLAB programme which specifies the protocol and decides, based on the detected lick signal, whether to apply MFB stimulation or apply time penalty. The MATLAB programme also triggers the optogenetic stimulus through the µLED control system which is separately operated using a LabVIEW programme. A National Instruments DAQ connects the lick detector, MFB stimulator and trigger signal for the µLED control system Arduino.

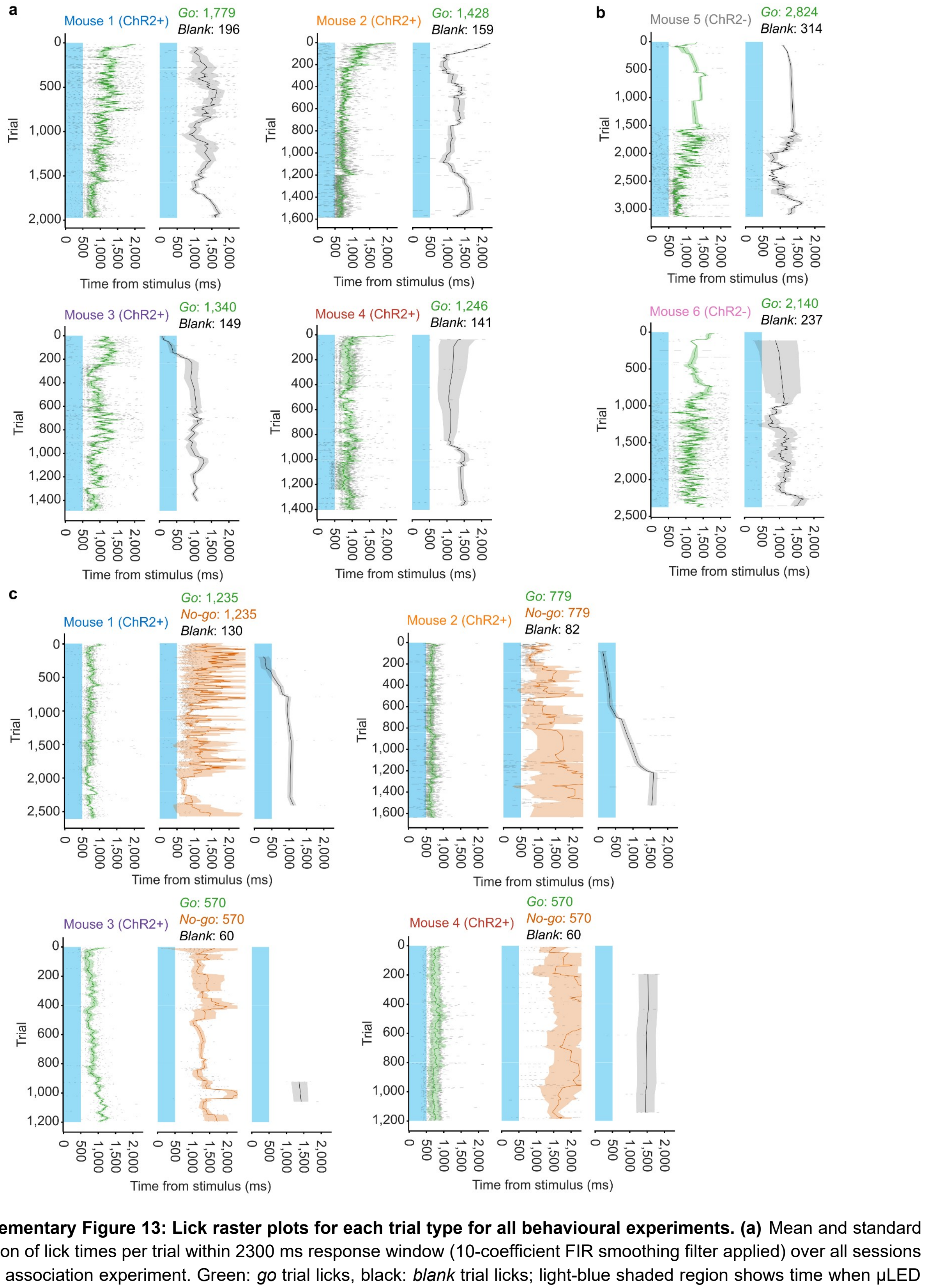

Supplementary Figure 13: Lick raster plots for each trial type for all behavioural experiments. (a) Mean and standard deviation of lick times per trial within 2300 ms response window (10-coefficient FIR smoothing filter applied) over all sessions for the association experiment. Green: *go* trial licks, black: *blank* trial licks; light-blue shaded region shows time when µLED was illuminated in a burst of 10 pulses (20 Hz, 50% duty cycle). **(b)** Same as (a) but for the control association experiment. **(c)** Same as (a) and (b) but for the discrimination experiment. Brown-red: *no-go* trial licks.

	Session	Session time (hr:min)	No. trials	Accum. trials	*Go* pattern	µLED irradiance (mW mm^{-2})	MFB voltage (V)	MFB duration (ms)
Mouse 1	1	1:26	300	300	P8N8 P9N9 P10N10	10	3.0	0.01
	2	1:18	205	505	P8N8 P9N9 P10N10	10	3.0	0.01
	3	1:23	233	738	P8N8 P9N9 P10N10	10	3.0	0.01
	4	1:39	400	1138	P1N1 P2N2 P3N3	10	2.4	0.01
	5	1:19	400	1538	P1N1 P2N2 P3N3	10	2.4	0.01
	6	0:44	400	1938	P1N4 P2N5 P3N6	8	2.4	0.01
Mouse 2	1	1:29	187	287	P1N8 P2N9 P3N10	10	1.8	0.01
	2	1:43	300	487	P1N8 P2N9 P3N10	10	1.8	0.01
	3	1:02	300	787	P1N8 P2N9 P3N10	10	1.7	0.01
	4	1:20	400	1187	P1N8 P2N9 P3N10	10	1.7	0.01
	5	1:12	400	1587	P1N8 P2N9 P3N10	10	1.7	0.01
Mouse 3	1	1:15	239	239	P1N1 P2N2 P3N3	10	2.0	0.001
	2	0:56	350	589	P1N1 P2N2 P3N3	10	2.0	0.001
	3	1:59	300	889	P1N1 P2N2 P3N3	10	2.0	0.001
	4	1:26	400	1289	P1N1 P2N2 P3N3	10	2.0	0.001
	5	0:34	200	1489	P3N2 P4N3 P5N4	6	2.0	0.001
Mouse 4	1	1:11	400	400	P1N1 P2N2 P3N3	10	2.6	0.01
	2	0:56	350	750	P1N1 P2N2 P3N3	10	2.6	0.01
	3	1:06	255	1005	P1N1 P2N2 P3N3	10	3.0	0.01
	4	1:16	400	1405	P2N3 P3N4 P4N5	8	2.6	0.01
Mouse 5	1	1:07	400	400	P8N1 P9N2 P10N3	8	2.2	0.001
	2	1:07	400	800	P8N1 P9N2 P10N3	8	2.2	0.001
	3	0:39	256	1056	P8N1 P9N2 P10N3	8	2.2	0.001
	4	1:53	600	1656	P8N1 P9N2 P10N3	8	2.6	0.001
	5	2:55	565	2221	P8N1 P9N2 P10N3	8	2.2	0.001
	6	1:30	210	2431	P8N1 P9N2 P10N3	8	2.0	0.001
	7	1:30	186	2617	P8N1 P9N2 P10N3	8	1.9	0.001
	8	1:11	521	3138	P8N1 P9N2 P10N3	8	1.7	0.001
Mouse 6	1	1:09	400	400	P1N8 P2N9 P3N10	6	1.8	0.01
	2	1:10	400	800	P1N8 P2N9 P3N10	6	1.8	0.01
	3	0:56	174	974	P1N8 P2N9 P3N10	6	1.8	0.01
	4	1:46	130	1104	P1N8 P2N9 P3N10	6	1.8	0.01
	5	4:18	225	1329	P1N8 P2N9 P3N10	6	1.9	0.01
	6	2:55	200	1529	P1N8 P2N9 P3N10	6	1.6	0.01
	7	2:26	228	1757	P1N8 P2N9 P3N10	6	1.2	0.01
	8	3:47	600	2357	P1N8 P2N9 P3N10	6	1.5	0.01

Supplementary Table 3: Summary of behavioural association experiment parameters.

	Session	Session time (hr:min)	No. trials	Accum. trials	*Go* pattern	*No-go* pattern	µLED irradiance (mW mm^{-2})	MFB voltage (V)	MFB duration (ms)
Mouse 1	1	1:26	600	600	P1N4 P2N5 P3N6	P8N8 P9N9 P10N10	8	2.4	0.01
	2	1:17	400	1000	P1N4 P2N5 P3N6	P8N8 P9N9 P10N10	8	2.4	0.01
	3	1:17	400	1400	P1N4 P2N5 P3N6	P8N8 P9N9 P10N10	8	2.3	0.01
	4	1:19	400	1800	P1N4 P2N5 P3N6	P8N8 P9N9 P10N10	8	2.3	0.01
	5	1:07	400	2200	P1N4 P2N5 P3N6	P8N8 P9N9 P10N10	8	2.2	0.01
	6	1:04	400	2600	P1N4 P2N5 P3N6	P8N8 P9N9 P10N10	8	2.2	0.01
Mouse 2	1	0:53	280	280	P1N8 P2N9 P3N10	P5N1 P6N2 P7N3	10	1.7	0.01
	2	0:50	280	560	P1N8 P2N9 P3N10	P5N1 P6N2 P7N3	10	1.7	0.01
	3	0:49	280	840	P1N8 P2N9 P3N10	P5N1 P6N2 P7N3	8	1.7	0.01
	4	0:43	400	1240	P1N8 P2N9 P3N10	P5N1 P6N2 P7N3	8	1.7	0.01
	5	0:43	400	1640	P1N8 P2N9 P3N10	P5N1 P6N2 P7N3	8	1.7	0.01
Mouse 3	1	1:11	400	400	P3N2 P4N3 P5N4	P8N8 P9N9 P10N10	6	2.0	0.001
	2	1:06	400	800	P3N2 P4N3 P5N4	P8N8 P9N9 P10N10	5	1.8	0.001
	3	1:09	400	1200	P3N2 P4N3 P5N4	P8N8 P9N9 P10N10	5	1.8	0.001
Mouse 4	1	1:11	400	400	P2N3 P3N4 P4N5	P8N8 P9N9 P10N10	6	2.6	0.01
	2	1:10	400	800	P2N3 P3N4 P4N5	P8N8 P9N9 P10N10	5	2.6	0.01
	3	1:05	400	1200	P2N3 P3N4 P4N5	P8N8 P9N9 P10N10	5	2.6	0.01

Supplementary Table 4: Summary of behavioural discrimination experiment parameters.

Association				Discrimination		
go + / blank +	go + / blank -	blank + / blank -	go - / blank -	go / no-go	go / blank	no-go / blank
0.00004	0.00027	0.08264	0.08264	0.00005	0.000003	0.05651

Supplementary Table 5: p values for statistical tests in Fig. 5f. Independent two-sample, single-tailed Student's t-test with alt. hypothesis: go accuracy > no-go or go accuracy > blank accuracy and/or ChR2+ accuracy > ChR2- accuracy. ChR2+: n = 4 mice, ChR2-: n = 2 mice.

a

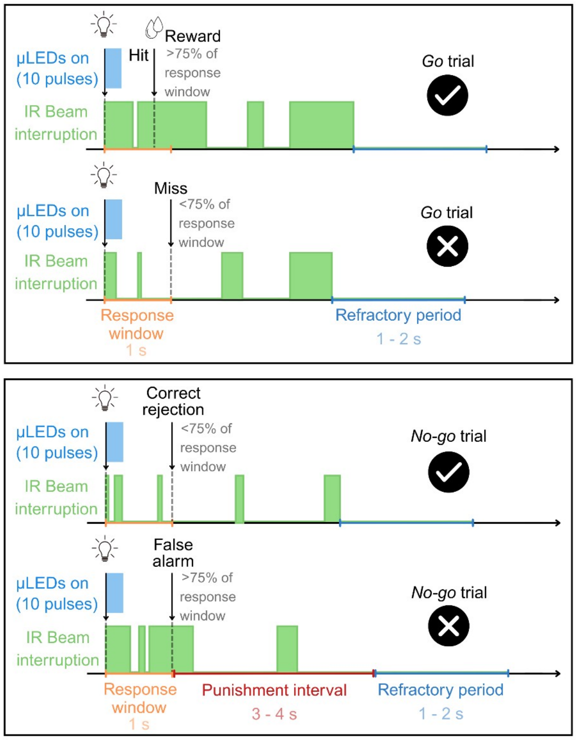

b

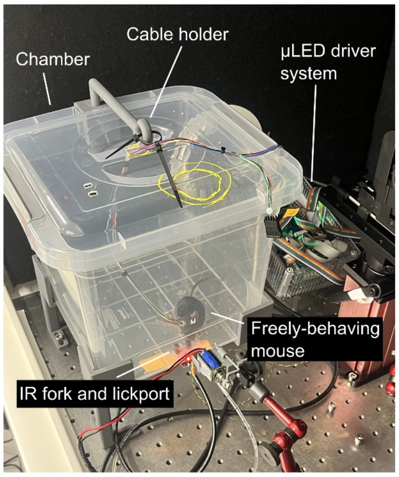

Supplementary Figure 14: Supplementary schematic on freely-behaving experiment. (a) Experimental timing diagrams for *go* trials (top) and *no-go* trials (bottom). Mouse should remain in infrared (IR) beam for a cumulative time of >75% of the 1 second response window on *go* trials, and leave the beam before the cumulative time reaches 75% of the response window on *no-go* trials. **(b)** Photograph of entire experimental setup consisting of behaviour chamber with IR beam and lickport, with cable holder affixed. µLED array is connected to control electronics through flexible cable and a freely-behaving mouse performing task is shown.

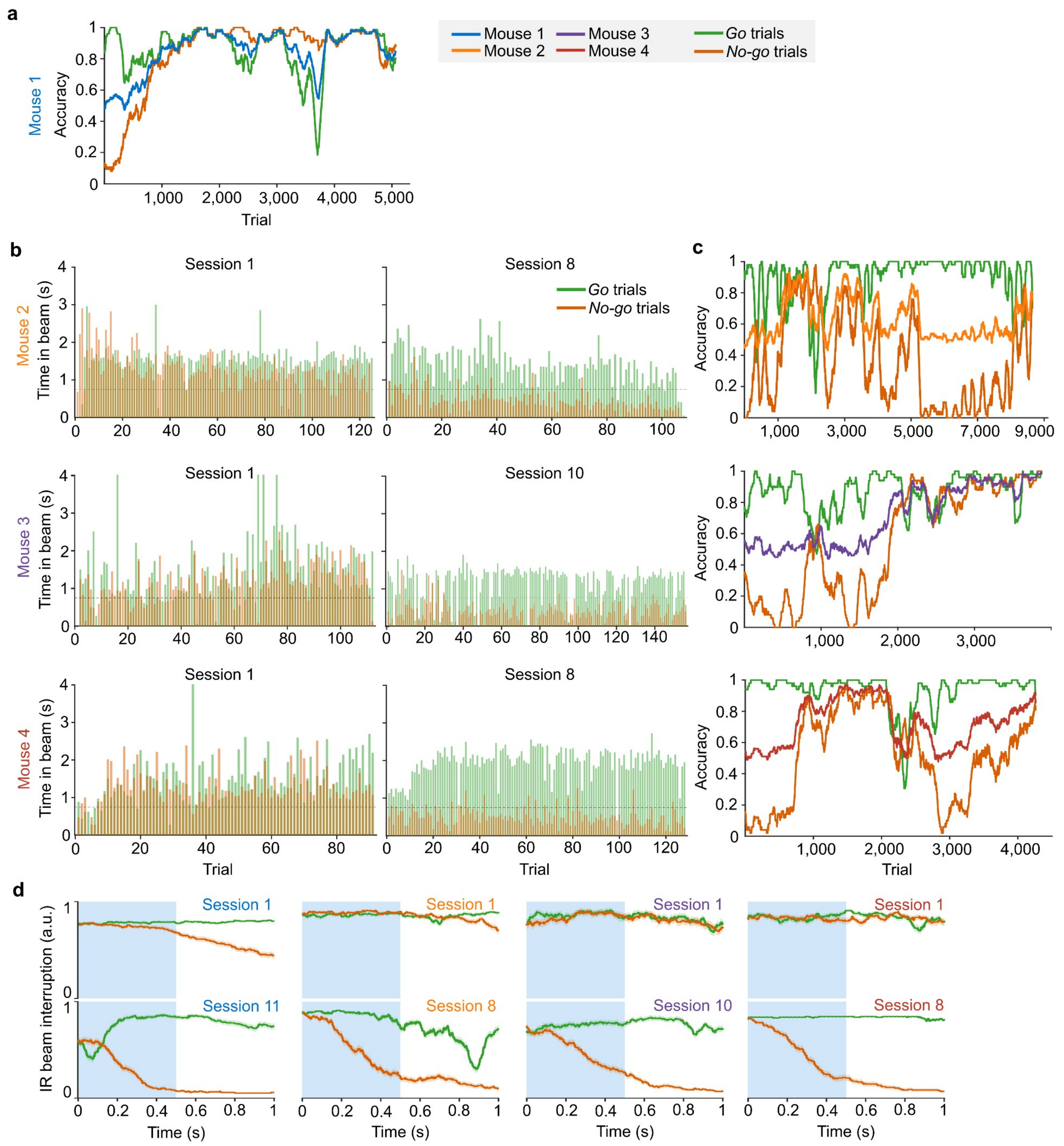

Supplementary Figure 15: Supplementary data analysis from freely-behaving discrimination experiments. (a) Accuracy (percentage of trials correct) for Mouse 1 across all sessions. Colours show accuracy for each trial type and average of trial types. **(b)** Time spent in the beam for each trial in the first session (left) and a session after which mouse has learned (right) for Mice 2, 3 and 4. **(c)** Accuracy (percentage of trials correct) for Mice 2, 3 and 4. **(d)** Intra-trial beam interruption over the course of the response window (mean and standard deviation for all *go* and *no-go* trials) between first session (top) and a session after which mouse has learned (bottom). 1: mouse present in IR beam; 0: mouse outwith IR beam.

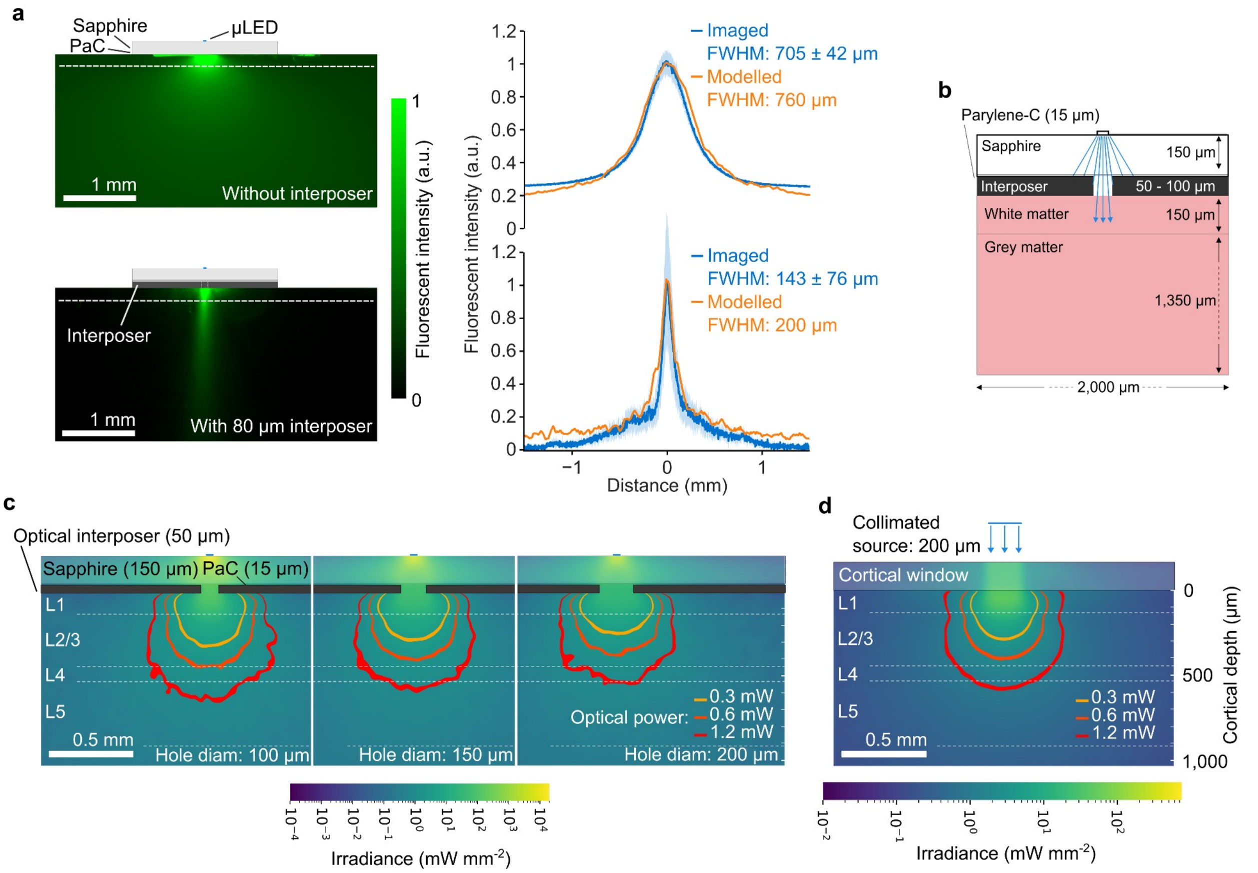

Supplementary Figure 16: Optical interposer (a) Emission profile of µLED with and without collimating interposer taken with SLR camera in fluorescein (left). Emission profile at distance of 150 µm from sapphire surface compared with cuts at equivalent distance from an optical model of fluorescence, with equivalent geometry. Error in image measurement given by n = 6 different camera integration times. **(b)** Schematic of optical model used to evaluate light propagation in brain tissue with collimating interposer. **(c)** Modelled optical profile for different optical interposer hole diameters; interposer thickness is 50 µm and cortical window is not present. Contours show 1 mW mm^{-2} threshold corresponding to equivalent optical power delivered to brain tissue as Fig. 4. µLED output power is scaled to deliver equivalent power to brain across models. **(d)** Modelled optical profile for perfect collimator, projecting light over 200 µm diameter area. Contours show 1 mW mm^{-2} threshold corresponding to equivalent optical power delivered to brain as (c).

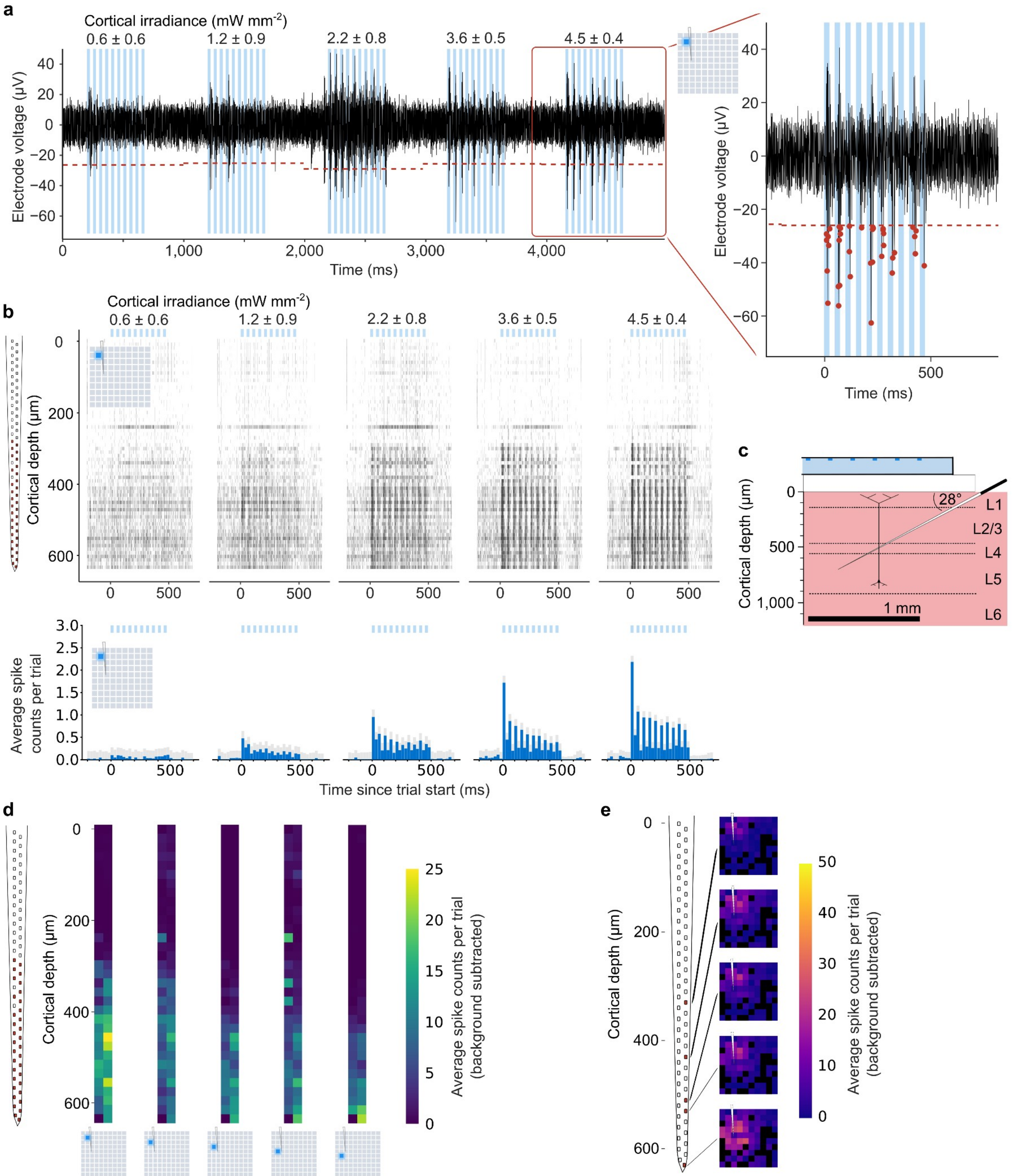

Supplementary Figure 17: Supplementary multi-unit analysis (MUA) using thresholding for Experiment 1. (a) Example of electrophysiology recording from a single electrode (filtered using bandpass FIR filter (cutoffs 300 Hz and 5.5 kHz), common median reference subtracted, and stimulation artifact blanked using rolling average). Each µLED is pulsed 10 times at 20 Hz, 50% duty cycle for 5 cortical irradiances (mean ± standard deviation) with corresponding µLED drive currents of 2.6, 4.2, 6.1, 8.3, 10.7 mA. Right plot shows threshold (5σ from signal mean) with a "spike" identified and counted if the signal passes below and back above the threshold. **(b)** Raster plot of counted spike times under stimulation from the same µLED as (a) for each electrode on the probe, accumulated across 50 trials (top). Peri-stimulus time histogram (PSTH) plot showing binned average spike counts per trial with mean of background subtracted, averaged over electrodes with time-locked response (bottom). **(c)** Schematic showing probe depth across cortical layers, for reference. **(d)** Average spike counts per trial (with background subtracted) for each electrode, as illuminated µLED position moves away from the probe. **(e)** Heat maps showing average spike counts per trial (with background subtracted) across all µLEDs individually illuminated, for selected electrode positions down the probe shank.

Experiment type	Experiment #	Device type	Encapsulation	Cortical window used
Electrophysiology	1, 2, 1-2, 3, 4, 5	Acute	PaC (7 µm)	Yes
Behaviour (head-fixed)	1	Chronic	PaC (15 µm)	No
	2	Chronic	PaC (15 µm)	No
	3	Chronic	PaC (15 µm)	No
	4	Chronic	PaC (15 µm)	No
	5	Chronic	PaC (15 µm)	No
	6	Chronic	PaC (15 µm)	No
Behaviour (freely-moving)	1	Chronic	PaC (15 µm)	No
	2	Chronic	PaC (30 µm) / silicone	No
	3	Chronic	PaC (15 µm)	No
	4	Chronic	PaC (15 µm)	No

Supplementary Table 6: Summary of devices and encapsulation schemes used in experiments; each row represents a different device. Note, the use of silicone in one of the encapsulation strategies covers only the device packaging and not the stimulation area; full details are given in the Methods section.
