# Supplementary material for: Chronically implantable μLED arrays for optogenetic cortical surface stimulation in mice
Source: Nat Commun. 2026 Jan 14;17:878. doi: 10.1038/s41467-025-68191-5 (PMC12827276; doi:10.1038/s41467-025-68191-5)
Supplement: Supplementary file 3 — Description of Additional Supplementary Files [file 41467_2025_68191_MOESM3_ESM.pdf]

Description of Additional Supplementary Files

Supplementary Data 1: Summary of freely-behaving experiment parameters.

Supplementary Movie 1: Patterned operation of μLED array.

Supplementary Movie 2: Freely-behaving mouse performing discrimination task.
