# Supplementary material for: Chronically implantable μLED arrays for optogenetic cortical surface stimulation in mice
Source: Nat Commun. 2026 Jan 14;17:878. doi: 10.1038/s41467-025-68191-5 (PMC12827276; doi:10.1038/s41467-025-68191-5)
Supplement: Supplementary file 7 — Reporting Summary [file 41467_2025_68191_MOESM7_ESM.pdf]

nature portfolio

Corresponding author(s): Keith Mathieson

Last updated by author(s): Nov 27, 2025

Reporting Summary

Nature Portfolio wishes to improve the reproducibility of the work that we publish. This form provides structure for consistency and transparency in reporting. For further information on Nature Portfolio policies, see our Editorial Policies and the Editorial Policy Checklist.

Statistics

For all statistical analyses, confirm that the following items are present in the figure legend, table legend, main text, or Methods section.

n/a	Confirmed	
☐	☒	The exact sample size (*n*) for each experimental group/condition, given as a discrete number and unit of measurement
☒	☐	A statement on whether measurements were taken from distinct samples or whether the same sample was measured repeatedly
☐	☒	The statistical test(s) used AND whether they are one- or two-sided<br>*Only common tests should be described solely by name; describe more complex techniques in the Methods section.*
☒	☐	A description of all covariates tested
☒	☐	A description of any assumptions or corrections, such as tests of normality and adjustment for multiple comparisons
☐	☒	A full description of the statistical parameters including central tendency (e.g. means) or other basic estimates (e.g. regression coefficient) AND variation (e.g. standard deviation) or associated estimates of uncertainty (e.g. confidence intervals)
☐	☒	For null hypothesis testing, the test statistic (e.g. *F*, *t*, *r*) with confidence intervals, effect sizes, degrees of freedom and *P* value noted<br>*Give P values as exact values whenever suitable.*
☒	☐	For Bayesian analysis, information on the choice of priors and Markov chain Monte Carlo settings
☒	☐	For hierarchical and complex designs, identification of the appropriate level for tests and full reporting of outcomes
☒	☐	Estimates of effect sizes (e.g. Cohen's *d*, Pearson's *r*), indicating how they were calculated

Our web collection on statistics for biologists contains articles on many of the points above.

Software and code

Policy information about availability of computer code

Data collection	Electrophysiology data was gathered using Intan RHX.<br>Spike sorting was carried out using Kilosort 2.5 and Phy, using Python 3.10.<br>LabVIEW 2023 was used to operate the electronic controller which was programmed using Arduino IDE.<br>MATLAB 2024 and LabVIEW 2023 were used to operate behavioural experiments.
Data analysis	Python 3.10 and the JupyterLab environment, with NumPy were used for all data analysis.<br>Figures with numerical data were plotted using the Plotly package.

For manuscripts utilizing custom algorithms or software that are central to the research but not yet described in published literature, software must be made available to editors and reviewers. We strongly encourage code deposition in a community repository (e.g. GitHub). See the Nature Portfolio guidelines for submitting code & software for further information.

Data

Policy information about availability of data

All manuscripts must include a data availability statement. This statement should provide the following information, where applicable:

- Accession codes, unique identifiers, or web links for publicly available datasets
- A description of any restrictions on data availability
- For clinical datasets or third party data, please ensure that the statement adheres to our policy

The raw data used to generate the figures in the article and supplementary material may be found at https://doi.org/10.15129/29d0a492-44fa-4294-a1e2-ee8de64fd958.
Due to large dataset sizes, requests for additional data including the in vivo electrophysiology or behavioural data, may be directed to the corresponding author.

Research involving human participants, their data, or biological material

Policy information about studies with human participants or human data. See also policy information about sex, gender (identity/presentation), and sexual orientation and race, ethnicity and racism.

Reporting on sex and gender	This study did not involve human participants.
Reporting on race, ethnicity, or other socially relevant groupings	See above.
Population characteristics	See above.
Recruitment	See above.
Ethics oversight	See above.

Note that full information on the approval of the study protocol must also be provided in the manuscript.

Field-specific reporting

Please select the one below that is the best fit for your research. If you are not sure, read the appropriate sections before making your selection.

☒ Life sciences ☐ Behavioural & social sciences ☐ Ecological, evolutionary & environmental sciences

For a reference copy of the document with all sections, see nature.com/documents/nr-reporting-summary-flat.pdf

Life sciences study design

All studies must disclose on these points even when the disclosure is negative.

Sample size	Number of animals used in electrophysiology experiments was based on standard sample sizes (4 - 6) from the literature involving in-vivo studies in mice.
Data exclusions	In electrophysiology experiments, one recording session was excluded from the main analysis due to the recording probe breakage prior to the experiment, which adversely affected the quality of the data. This is still included in the supplementary information.
Replication	Several mice were used in both electrophysiology and behavioural experiments, with similar craniotomy preparations and chronic implantations. Consistent results across animals were observed.
Randomization	All optogenetic and behavioral experiments employed pseudo-random stimulation protocols, i.e. the position of the illuminated microLED/ microLED pattern varied throughout the course of experiments and trial types were pseudo-randomized in behavioral experiments.
Blinding	Blinding was not relevant due as experimental and quantification procedures were automated (behavior, electrophysiology)

Reporting for specific materials, systems and methods

We require information from authors about some types of materials, experimental systems and methods used in many studies. Here, indicate whether each material, system or method listed is relevant to your study. If you are not sure if a list item applies to your research, read the appropriate section before selecting a response.

Materials & experimental systems

n/a	Involved in the study	
☐	☒	Antibodies
☒	☐	Eukaryotic cell lines
☒	☐	Palaeontology and archaeology
☐	☒	Animals and other organisms
☒	☐	Clinical data
☒	☐	Dual use research of concern
☒	☐	Plants

Methods

n/a	Involved in the study	
☒	☐	ChIP-seq
☒	☐	Flow cytometry
☒	☐	MRI-based neuroimaging

Antibodies

Antibodies used	- Anti-Iba1 antibody, monoclonal, Clone #: EPR16588, Catalogue #: ab178846 https://www.abcam.com/en-us/products/primary-antibodies/iba1-antibody-epr16588-ab178846
Validation	As per Abcam website "Abcam's high quality manufacturing and validation processes ensure Anti-Iba1 antibody [EPR16588] (ab178846) has high sensitivity and specificity alongside high lot-to-lot consistency and reproducibility."

Animals and other research organisms

Policy information about studies involving animals; ARRIVE guidelines recommended for reporting animal research, and Sex and Gender in Research

Laboratory animals	Optogenetic experiments were conducted on adult transgenic mice (Emx1-IRES-Cre x Ai27D). Control experiments were performed on adult transgenic mice (Emx1-Cre) that did not express any opsin.<br>For freely-behaving experiments only, mice were transfected with pAAV-CamKIIa-Chrmine-mScarlet-Kv2.1-WPRE (AAV8) Addgene number: 130991
Wild animals	The study did not involve wild animals.
Reporting on sex	Similar numbers of male and female mice were used throughout as standard practice. Sex-based analysis was not relevant in the study of recording neuronal and behavioural response to optogenetic stimulation of the auditory cortex.
Field-collected samples	This study did not involve samples that were collected from the field.
Ethics oversight	All experiments were performed in accordance with the French Ethical Committee (Direction Générale de la Recherche et de l'Innovation) and European legislation (2010/63/EU). Procedures were approved by the French Ministry of Education in Research after consultation with the ethical committee #59 (authorization number APAFIS#28757-2020122111048873).

Note that full information on the approval of the study protocol must also be provided in the manuscript.

Plants

Seed stocks	This study did not involve plants.
Novel plant genotypes	See above.
Authentication	See above.
